# Rational Engineering of Phenylalanine Accumulation in *Pseudomonas taiwanensis* to Enable High-Yield Production of *Trans*-Cinnamate

**DOI:** 10.3389/fbioe.2019.00312

**Published:** 2019-11-20

**Authors:** Maike Otto, Benedikt Wynands, Christoph Lenzen, Melanie Filbig, Lars M. Blank, Nick Wierckx

**Affiliations:** ^1^Institute of Bio- and Geosciences (IBG-1: Biotechnology), Forschungszentrum Jülich GmbH, Jülich, Germany; ^2^Institute of Applied Microbiology, Rheinisch-Westfälische Technische Hochschule (RWTH) Aachen University, Aachen, Germany

**Keywords:** *Pseudomonas*, metabolic engineering, *trans*-cinnamic acid, L-phenylalanine, rational engineering, glycerol, glucose

## Abstract

Microbial biocatalysis represents a promising alternative for the production of a variety of aromatic chemicals, where microorganisms are engineered to convert a renewable feedstock under mild production conditions into a valuable chemical building block. This study describes the rational engineering of the solvent-tolerant bacterium *Pseudomonas taiwanensis* VLB120 toward accumulation of L-phenylalanine and its conversion into the chemical building block *t*-cinnamate. We recently reported rational engineering of *Pseudomonas* toward L-tyrosine accumulation by the insertion of genetic modifications that allow both enhanced flux and prevent aromatics degradation. Building on this knowledge, three genes encoding for enzymes involved in the degradation of L-phenylalanine were deleted to allow accumulation of 2.6 mM of L-phenylalanine from 20 mM glucose. The amino acid was subsequently converted into the aromatic model compound *t*-cinnamate by the expression of a phenylalanine ammonia-lyase (PAL) from *Arabidopsis thaliana*. The engineered strains produced *t*-cinnamate with yields of 23 and 39% Cmol Cmol^−1^ from glucose and glycerol, respectively. Yields were improved up to 48% Cmol Cmol^−1^ from glycerol when two enzymes involved in the shikimate pathway were additionally overexpressed, however with negative impact on strain performance and reproducibility. Production titers were increased in fed-batch fermentations, in which 33.5 mM *t*-cinnamate were produced solely from glycerol, in a mineral medium without additional complex supplements. The aspect of product toxicity was targeted by the utilization of a streamlined, genome-reduced strain, which improves upon the already high tolerance of *P. taiwanensis* VLB120 toward *t*-cinnamate.

## Introduction

*Trans*-cinnamate is an aromatic compound naturally occurring in plants, where it serves as central intermediate for the biosynthesis of a large number of substances, including coumarins, flavonoids, and phenylpropanoids (Chemler and Koffas, 2008; Vogt, 2010). It is widely used in industry for flavoring, pharmaceuticals, and cosmetics (Fausta et al., 1999; De et al., 2011; Vargas-Tah and Gosset, 2015) and can serve as precursor for bio-based drop-in chemicals such as styrene (McKenna and Nielsen, 2011) or for value-added chemicals such as the stilbene pinosylvin (van Summeren-Wesenhagen and Marienhagen, 2015). Commercial production currently happens from petroleum-based, non-renewable feedstocks in processes that demand high amounts of energy and release toxic by-products (Bruckner, 2010; Tietze et al., 2015). This comes along with increasing apprehension on global climate change and depleting aromatic fossil resources. Hence, there are both environmental and economic drivers for alternative synthesis routes.

Microbial production by whole-cell biocatalysis represents a less environmentally demanding alternative to the common and well-established petrochemical processes (Hatti-Kaul et al., 2007; Becker and Wittmann, 2012; Cho et al., 2015; Kallscheuer et al., 2019). Herein, microbes can be supplied with renewable feedstocks (e.g., glucose, glycerol, or lignin, Kohlstedt et al., 2018; Johnson et al., 2019), and metabolic conversion enables the synthesis of diverse products under mild production conditions, either via native enzymes (Hosseinpour Tehrani et al., 2019) or by heterologous expression of foreign genes (Kallscheuer et al., 2019). In plants, *t*-cinnamate is formed through deamination of the amino acid L-phenylalanine by phenylalanine ammonia-lyase (PAL) (Cochrane et al., 2004; Huang et al., 2010), and can be further converted into the *cis*-isoform by photoisomerization (Salum and Erra-Balsells, 2013). While only the *trans*-isoform is involved in biosynthesis pathways in plants (Salum and Erra-Balsells, 2013), *cis*-cinnamate displays higher anti-bacterial activities (Chen et al., 2011). The heterologous synthesis of this enzyme in various microorganisms, including *Escherichia coli* (Vargas-Tah et al., 2015; Bang et al., 2018) or *Streptomyces lividans* (Noda et al., 2011) enabled *t*-cinnamate production. Product toxicity is a limiting factor for the efficiency of many microbial production process with these hosts (McKenna and Nielsen, 2011). From a biochemical engineering perspective, it is important to “begin with the end in mind” when developing microbial production strains (Straathof et al., 2019), and product toxicity is one important aspect for this.

Bacteria of the genus *Pseudomonas* are considered as promising alternative host to produce aromatics such as *t*-cinnamate. Their robust growth behavior and metabolic versatility recently enabled the synthesis of many different industrially relevant compounds, including a variety of aromatics (Nijkamp et al., 2007; Kuepper et al., 2015; Wynands et al., 2018), rhamnolipids (Tiso et al., 2017), terpenes (Mi et al., 2014), or prodiginines (Domröse et al., 2015). This is in particular due to their high stress tolerance, which has been extensively investigated in the past (Kusumawardhani et al., 2018). Pseudomonads are able to thrive under both endogenous and exogenous oxidative stress, enabled by their particular central carbon metabolism architecture (Chavarría et al., 2013; Nikel et al., 2015). Furthermore, they are equipped with a variety of native tolerance mechanisms that allow growth in the presence of highly toxic compounds (Sardessai and Bhosle, 2002; Segura et al., 2012). For this, Pseudomonads are able to adapt the composition of the inner and outer membrane to lower the permeability for substrates and they can increase their production of energy to fuel energy-consuming tolerance mechanisms (Isken and de Bont, 1998; Ramos et al., 2002; Belda et al., 2016). Some strains of *Pseudomonas* additionally express solvent efflux pumps that can actively extrude toxic compounds from the inner membrane (Kieboom et al., 1998) and degrade solvents such as toluene (Ramos et al., 2002) or styrene (Velasco et al., 1998). Additional transporters confer higher resistance to further aromatic molecules like *p*-hydroxybenzoate or *p*-coumarate (Verhoef et al., 2010; Calero et al., 2018). The native capabilities of *Pseudomonas* species thus form a strong basis for a biocatalytic process for *t*-cinnamate synthesis by expanding process options due to reduced product toxicity.

To further exploit its potential as industrial production strain, we recently enhanced the bioprocess features of the solvent-tolerant strain *Pseudomonas taiwanensis* VLB120 by successive feature reduction (Wynands et al., 2019). The deletion of redundant genomic elements such as proviral segments, genes for biofilm formation and flagella expression and the megaplasmid pSTY resulted in strains with 15% enhanced growth rates and increased biomass yields, thereby improving the overall performance of the strain under bioprocess conditions. In addition, genes encoding for the efflux pump TtgGHI located on the pSTY plasmid were re-integrated into the chromosome of the genome-reduced chassis (GRC) strains to maintain tolerance enabled by this efflux pump. These modifications, in combination with inherent tolerance traits, make the engineered strains promising hosts to efficiently synthesize aromatic compounds like *t*-cinnamate, as well as potential hydrophobic derivatives such as styrene or stilbenes.

The potential of *Pseudomonas* species for *t*-cinnamate production has been demonstrated in a strain of *P. putida* S12 by the expression of PAL from *Rhodosporidium toruloides* (Nijkamp et al., 2005). Here, enhanced precursor supply was realized by mutagenesis and subsequent selection on the toxic analog *m*-fluoro-phenylalanine, leading to the production of 5 mM *t*-cinnamate with a yield of 6.7% Cmol Cmol^−1^. However, although genomic analysis of this strain provided leads to enhance the flux through the shikimate pathway in *P. taiwanensis* VLB120 (Wynands et al., 2018), the genetic rearrangements enabling the enhanced flux specifically toward L-phenylalanine have not been determined. We recently reported a study on phenol production from L-tyrosine in *P. taiwanensis* VLB120, where 22 mutations in 50 different strains delivered a comprehensive insight on the rational engineering of L-tyrosine accumulation (Wynands et al., 2018). In the most productive strain, five genes involved in the degradation of aromatic intermediates and the gene *pykA* were deleted to increase the precursor supply for the synthesis of aromatic amino acids. The flux through the shikimate pathway was further enhanced by genomic modifications of genes at their native chromosomal locus resulting in a strain able to accumulate up to 2.8 mM of L-tyrosine from 20 mM glucose.

The aim of this study was to build on this knowledge to redirect the enhanced flux to L-tyrosine in *P. taiwanensis* GRC3 Δ5Δ*pykA*-tap (Wynands, 2018) toward L-phenylalanine, given the close relation of their biosynthesis pathways, and thereby expanding the potential product range. Furthermore, the superior tolerance of the streamlined *P. taiwanensis* GRC toward high concentrations of *t*-cinnamate was assessed. Routes involved in L-phenylalanine degradation were deleted, leading to L-phenylalanine accumulation. Titers were further increased by an additional overexpression of feedback-resistant versions of the bottleneck enzymes AroG and PheA. Heterologous expression of a PAL from *Arabidopsis thaliana* in the engineered strains then enabled the production of *t*-cinnamate. In a completely minimal medium, the plasmid-free chassis strains produced up to 6.3 mM of *t*-cinnamate from glycerol, which corresponds to a yield of 48% Cmol Cmol^−1^. Cultivation in fed-batch fermentations resulted in titers of around 33.5 mM, which is the highest reported titer of microbially produced *t*-cinnamate in a cultivation medium without complex supplements. The engineered strains can further serve as efficient platform to produce various products from L-phenylalanine and *t*-cinnamate, including styrene, benzoate, or plant polyphenols such as pinosylvin.

## Materials and Methods

### Bacterial Strains, Plasmids, and Cultivation Conditions

Strains and plasmids used in this study can be found in Tables 1, 2. For cloning purposes, *E. coli* and *Pseudomonas* cells were cultivated at 37 or 30°C, respectively, either in liquid LB medium containing 5 g L^−1^ sodium chloride or on solid LB agar plates [additionally containing 1.5% (w/v) agar]. After tri- or four-parental mating procedures, Pseudomonads were isolated on cetrimide agar (Sigma Aldrich) plates supplemented with 10 mL L^−1^ glycerol. Kanamycin (50 μg mL^−1^) or gentamicin (20 μg mL^−1^) was added to cultures or plates when necessary.

**Table 1:** Plasmids.

**Plasmid**	**Description**	**References**
pEMG	Km^R^, oriR6K, lacZα with two flanking I-SceI sites	Martínez-García and de Lorenzo, 2011
pSW-2	Gm^R^, oriRK2, *xylS*, Pm → I-SceI	Martínez-García and de Lorenzo, 2011
pEMG-*pobA*	pEMG bearing flanking sequences of *pobA, pobA* deletion delivery vector	Wynands et al., 2018
pEMG-*hpd*	pEMG bearing flanking sequences of *hpd, hpd* deletion delivery vector	Wynands et al., 2018
pEMG-*quiC*	pEMG bearing flanking sequences of *quiC, quiC* deletion delivery vector	Wynands et al., 2018
pEMG-*quiC1*	pEMG bearing flanking sequences of *quiC1, quiC1* deletion delivery vector	Wynands et al., 2018
pEMGu-PVLB_13075	pEMGu bearing flanking sequences of *quiC2, quiC2* deletion delivery vector	Wynands et al., 2018
pEMG-*pykA*	pEMG bearing flanking sequences of *pykA, pykA* deletion delivery vector	Wynands et al., 2018
pEMGu-*trpE*^P290S^	pEMGu bearing flanking sequences of *trpE*^P290S^, P290S substitution delivery vector	Wynands et al., 2018
pEMGg-*aroF-1*^P148L^	pEMGg bearing flanking sequences of *aroF-1*^P148L^, P148L substitution delivery vector	Wynands et al., 2018
pEMGg-*pheA*^T310I^	pEMGg bearing flanking sequences of *pheA*^T310I^, T310I substitution delivery vector	Wynands et al., 2018
pEMG-*phhAB*	pEMG bearing flanking sequences of *phhAB, phhAB* deletion delivery vector	This study
pEMG-*katG*	pEMG bearing flanking sequences of *katG, katG* deletion delivery vector	This study
pEMG-PVLB_10925	pEMG bearing flanking sequences of PVLB_10925, PVLB_10925 deletion delivery vector	This study
pBG_14d_-*msfgfp*	Km^R^ Gm^R^, ori R6K, Tn7L and Tn7R extremes, P*_14*d*_*-BCD2–*msfgfp* fusion	Zobel et al., 2015
pBG_14f_-*msfgfp*	Km^R^ Gm^R^, ori R6K, Tn7L and Tn7R extremes, P_14f_-BCD2–*msfgfp* fusion	Zobel et al., 2015
pBG_14g_-*msfgfp*	Km^R^ Gm^R^, ori R6K, Tn7L and Tn7R extremes, P_14g_-BCD2–*msfgfp* fusion	Zobel et al., 2015
pBG_14d_-a*roG*^fbr^*-pheA*^T310I^	Km^R^ Gm^R^, ori R6K, Tn7L and Tn7R extremes, P*_14*d*_*-BCD2–a*roG*^fbr^ fusion, a*roG*^fbr^ from *E.coli* K12 W3110, p*heA*^T310I^ from *P. putida* S12palM12	This study
pBG_14g_*AtPAL- aroG*^fbr^* -pheA*^T310^	Km^R^ Gm^R^, ori R6K, Tn7L and Tn7R extremes, P_14g_-BCD2–a*roG*^fbr^ fusion, a*roG*^fbr^ from *E.coli* K12 W3110, p*heA*^T310I^ from *P. putida* S12palM12, *PAL2* from *A. thaliana* codon optimized for *P. taiwanensis* VLB120	This study
pBG_14f_-*AtPAL*	Km^R^ Gm^R^, ori R6K, Tn7L and Tn7R extremes, P_14f_-BCD2–At-PAL fusion, *PAL2* from *A. thaliana* codon optimized for *P. taiwanensis* VLB120	This study

**Table 2:** Bacterial strains.

**Strain**	**Description**	**References**
**E. coli**		
DH5α λpir	*E44, ΔlacU169 (ΦlacZΔM15), recA1, endA1, hsdR17, thi-1, gyrA96, relA1, λpir phage lysogen;* host for oriV(R6K) vectors	de Lorenzo and Timmis, 1994
PIR2	*F^−^Δlac169 rpoS(Am) robA1 creC510 hsdR514 endA reacA1 uidA(ΔMlui)::pir*, host for oriV(R6K) vectors	Invitrogen
HB101 pRK2013	*F^−^ mcrB mrr hsdS20(rB^−^ mB ^−^) recA13 leuB6 ara^−^14 proA2 lacY1 galK2 xyl-5 mtl-1 rpsL20(Sm^*R*^) gln V44 λ^−^* bearing pRK2013	Boyer and Roulland-Dussoix, 1969
DH5α λpir pTNS1	DH5α λpir bearing plasmid pTNS1	de Lorenzo Lab
***P. taiwanensis***		
VLB120	Wildtype	Panke et al., 1998
VLB120ΔpSTY	ΔpSTY	Wynands et al., 2019
GRC1	ΔpSTY, Δprophage1/2, Δprophage3, Δprophage4, Δflag1, Δflag2, Δlap1, Δlap2, Δlap3	Wynands et al., 2019
GRC2	ΔpSTY, Δprophage1/2::*ttgGHI*, Δprophage3, Δprophage4, Δflag1, Δflag2, Δlap1, Δlap2, Δlap3	Wynands et al., 2019
GRC3	ΔpSTY, Δprophage1/2::*VWGHI*, Δprophage3, Δprophage4, Δflag1, Δflag2, Δlap1, Δlap2, Δlap3	Wynands et al., 2019
GRC3 Δ5Δ*pykA*-tap	GRC3 with Δ*pobA*, Δ*hpd*, Δ*quiC*, Δ*quiC1*, Δ*quiC2*, Δ*pykA, trpE*^P290S^, *aroF-1*^P148L^, *pheA*^T310I^	Wynands, 2018
GRC3 Δ5Δ*pykA*-tap Δ*phhAB*	GRC3 Δ5Δ*pykA*-tap with Δ*phhAB*	This study
GRC3 Δ5Δ*pykA*-tap Δ*phhABΔkatG*	GRC3 Δ5Δ*pykA*-tap with Δ*phhAB*, Δ*katG*	This study
GRC3 Δ5Δ*pykA*-tap Δ*phhAB*ΔPVLB_10925	GRC3 Δ5Δ*pykA*-tap with Δ*phhAB*, ΔPVLB_10925	This study
GRC3 Δ8Δ*pykA*-tap	GRC3 Δ5Δ*pykA*-tap with Δ*phhAB*, Δ*katG*, ΔPVLB_10925	This study
GRC3 Δ8Δ*pykA*-tap *attTn7*::P_14g_*AtPAL- aroG*^fbr^* -pheA*^T310^	GRC3 Δ8Δ*pykA*-tap with P_14g_*AtPAL- aroG*^fbr^* -pheA*^T310^ chromosomally integrated at site *attTn7*	This study
GRC3 Δ8Δ*pykA*-tap *attTn7*::P_14f_*AtPAL*	GRC3 Δ8Δ*pykA*-tap with P_14f_*AtPAL* chromosomally integrated at site *attTn7*	This study
GRC3 Δ5Δ*pykA*-tap *attTn7*::P_14d_-a*roG*^fbr^*-pheA*^T310^	GRC3 Δ5Δ*pykA*-tap with P_14d_-a*roG*^fbr^*-pheA*^T310I^ chromosomally integrated at site *attTn7*	This study
GRC3 Δ5Δ*pykA*-tap Δ*phhAB attTn7*::P_14d_-a*roG^*fbr*^-pheA^*T*310^*	GRC3 Δ5Δ*pykA*-tapΔ*phhAB* with P_14d_-a*roG^*fbr*^-pheA^*T*310^* chromosomally integrated at site *attTn7*	This study
GRC3 Δ5Δ*pykA*-tap Δ*phhABΔkatG attTn7*::P_14d_-a*roG^*fbr*^-pheA^*T*310^*	GRC3 Δ5Δ*pykA*-tapΔ*phhABΔkatG* with P_14d_-a*roG^*fbr*^-aheA^*T*310^* chromosomally integrated at site *attTn7*	This study
GRC3 Δ5Δ*pykA*-tap Δ*phhAB*ΔPVLB_10925 *attTn7*::P_14d_-a*roG^*fbr*^-pheA^*T*310^*	GRC3 Δ5Δ*pykA*-tapΔ*phhABΔPVLB_10925* with P_14d_-a*roG^*fbr*^-pheA^*T*310^* chromosomally integrated at site *attTn7*	This study
GRC3 Δ8Δ*pykA*-tap *attTn7*::P_14d_-a*roG^*fbr*^-pheA^*T*310^*	GRC3 Δ5Δ*pykA*-tapΔ*phhABΔkatGΔPVLB_10925* with P_14d_-a*roG^*fbr*^-pheA^*T*310^* chromosomally integrated at site *attTn7*	This study

During production and toxicity experiments in shake flasks and well plates, liquid cultures of *P. taiwanensis* were grown in mineral salt medium (MSM) adapted from Hartmans et al. (1989) at pH 7.0 without the addition of antibiotics. The medium's standard phosphate buffer capacity was increased to 5-fold (111.5 mM K_2_HPO_4_ and 68 mM NaH_2_PO_4_). Glucose or glycerol were added as sole carbon source at indicated concentrations. Main cultures were inoculated at an OD_600_ of 0.2, from seed cultures grown in MSM containing glucose as carbon source. Production experiments were performed in 500 mL Erlenmeyer flasks with a culture volume of 50 mL, which were cultivated in a rotary shaker with a frequency of 200 rpm and a throw of 50 mm. Toxicity assays were performed in 96-round low-well plates with a filling volume of 200 μL in the System Duetz® cultivation system (EnzyScreen, Leiden, Netherlands) with a shaking frequency of 225 rpm. OD_600_ values were calculated from the measured green values using a calibration. Phenylalanine accumulation was analyzed in 24-square deep-well plates with a filling volume of 5 mL and a shaking frequency of 225 rpm, and growth was monitored in the Growth profiler® system.

### Plasmid Construction and Genomic Modification

For plasmid construction, DNA fragments were PCR amplified using the Q5 high-fidelity polymerase (New England Biolabs, New Ipswich, USA) with corresponding overhangs to enable subsequent Gibson assembly (Gibson et al., 2009) with the HiFi DNA assembly master mix (New England Biolabs, New Ipswich, USA). Primers were ordered from Eurofins Genomics (Ebersberg, Germany). The gene sequences for *aroG*^fbr^ (from *E. coli* K12 W3110) and AtPAL (from *A. thaliana*) were codon-optimized for *P. taiwanensis* VLB120 using the online tool OPTIMIZER (Puigbo et al., 2007) and ordered as synthetic DNA fragments from Thermo Fisher Scientific (Waltham, USA). The gene *pheA*^T310I^ was amplified from genomic DNA of strain *P. putida* S12palM12. pEMG-based plasmids were transformed into *E. coli* DH5α λpir cells, pBG-based plasmids were transformed into *E. coli* PIR2. Correct plasmid assembly was verified by Sanger sequencing performed by Eurofins Genomics (Ebersberg, Germany). Integration at the *attTn7*-site was achieved by patch-mating of the *E. coli* donor strain holding the respective pBG-plasmid, the helper strain *E. coli* HB101 pRK2013, DH5α λpir pTNS1 providing the required transposase and the recipient *Pseudomonas taiwanensis* strain as described by Wynands et al. (2018). After the mating procedure, *Pseudomonas* were isolated on Cetrimide agar containing gentamicin and correct integration was confirmed by colony PCR using the OneTaq Quick-Load Master Mix (New England Biolabs, New Ipswich, USA).

Genomic deletions and point mutations were realized using the I-SceI-based method developed by Martínez-García and de Lorenzo (2011) using a streamlined protocol adapted by Wynands et al. (2018). Successful deletions were verified by colony PCR using the OneTaq quick-Load Master Mix (New England Biolabs, New Ipswich, USA).

### Fed-Batch in Controlled Bioreactors

Fermentations were carried out in fed-batch operation mode in DASbox® mini-bioreactors using the DASware control software (Eppendorf, Hamburg, Germany). The reactors were set up of 385 ml glass vessels, two Rushton-type impellers driven by direct overhead drives, feeding lines for acid, base, and carbon source, a temperature sensor, an EasyFerm Plus K8 120 pH-sensor (Hamilton Company, Reno, NV, USA) and an InPro® 6800 series O_2_ sensor (Mettler-Toledo International Inc., Columbus, OH, USA). The starting volume was 100 mL and the temperature was maintained at 30°C. Gas supply was provided via headspace with a starting gas flow rate of 6 sL h^−1^. The initial agitation frequency was 500 rpm. The dO_2_ was controlled with a cascade, keeping the concentration of diluted oxygen at 35% by first increasing the agitation speed up to 1,200 rpm, then increasing the oxygen concentration in the air supply to a maximum of 80% and subsequently increasing the gas flow rate. pH 7 was maintained by automatic addition of 5% NH_3_ or 1 M H_2_SO_4_. Cells were grown in MSM according to Hartmans et al. (1989), where the addition of mineral salts stock was increased by 2-fold (MgCl_2_.6H_2_O 0.2 g L^−1^, ZnSO_4_.7H_2_O 0.004 g L^−1^, CaCl_2_.2H_2_O 0.002 g L^−1^, FeSO_4_.7H_2_O 0.01 g L^−1^, Na_2_MoO_4_.2H_2_O 0.0004 g L^−1^, CuSO_4_.5H_2_O 0.0004 g L^−1^, CoCl_2_.6H_2_O 0.0008 g L^−1^, MnCL_2_.2H_2_O 0.002 g L^−1^). The initial batch medium contained either 20 mM of glucose or 40 mM of glycerol as sole carbon source. The reactors were operated in a dO_2_-controlled fed-batch mode. When glucose or glycerol was depleted, the dO_2_ signal rapidly increased as a result of metabolic arrest. A dO_2_ signal >70 triggered a feed pump, resulting in a feed shot of 5 mM of glucose or 10 mM of glycerol per trigger initiation.

### Analytical Methods

Optical densities of liquid cultures were measured at 600 nm using an Ultrospec 10 Cell Density Meter (GE Healthcare, Illinois, USA).

Samples taken from the cultures were centrifuged at 13,000 rpm for 2–5 min and the supernatant was analyzed by HPLC. Aromatics quantification was performed using a Beckman System Gold 126 Solvent Module with a 168 diode 201 array detector (Beckman Coulter, Brea, USA) and an ISAspher 100-5 C18 BDS reversed-phase 202 column (ISERA, Düren, Germany) at 30°C and a flow rate of 0.8 mL min^−1^. Elution took place with a gradient starting at 90% H_2_O containing 0.1% (v/v) TFA and 10% methanol. This ratio was held for 2 min, followed by gradual increase to 100% methanol over the course of 8 min. After 2 min at 100% methanol, initial ratios were reached again within 1 min and held constant for further 2 min. UV detection was conducted at a wavelength of 245 nm. L-phenylalanine and L-tyrosine quantification shown in Figure 3 was performed with the same column and elution setup as mentioned before, in a Dionex Ultimate 3000 HPLC system, where detection took place with a Corona Veo charged aerosol detector (Thermo Fisher Scientific, Waltham, MA, USA). Glucose, gluconate and glycerol analysis was performed using a Beckman System Gold 126 Solvent Module with a System Gold 166 UV-detector (Beckman Coulter, Brea, USA) and Smartline RI Detector 2300 (KNAUER, Berlin, Germany) on a MetabAAC column (ISERA, Düren, Germany). Elution took place with 5 mM H_2_SO_4_ at an isocratic flow of 0.5 mL min^−1^ and a temperature of 30°C for 20 min. Glucose and glycerol were analyzed using the RI detector, gluconate concentrations were determined with the UV detector at a wavelength of 210 nm. Due to co-elution of glucose and gluconate, glucose concentrations were determined by subtraction of the gluconate concentration.

## Results and Discussion

### Enhanced *Trans*-Cinnamate Tolerance of Streamlined *P. taiwanensis* Chassis Strains

Product toxicity vastly influences the efficiency of a microbial production process. Cellular stress induced by toxic compounds leads to decreased production rates or complete growth arrest, thereby limiting feasible product titers and yields (Sardessai and Bhosle, 2002; McKenna and Nielsen, 2011; Kusumawardhani et al., 2018). Compared to hydrophobic aromatics such as styrene, *t*-cinnamate is mildly toxic at neutral pH and it exhibits a different mode of action. Increasing concentrations of *t*-cinnamate lead to enhanced osmotic stress in cell cultures, but will also cause the inhibition of specific enzymes and disturb cellular processes, resulting in growth defects (Olasupo et al., 2003; Guzman, 2014). Growth of *E. coli*, for example, is heavily impaired at concentrations above 2.7 mM (400 mg L^−1^) and completely inhibited at >5.4 mM (800 mg L^−1^) when *t*-cinnamate is added at the beginning of cultivation (Olasupo et al., 2003; McKenna and Nielsen, 2011). This necessitates the addition of expensive complex supplements such as yeast extract or casamino acids or a biotransformation approach with late PAL induction at high cell densities to allow for sufficient accumulation of the precursor L-phenylalanine (Noda et al., 2011; Bang et al., 2018).

**Figure 1:**
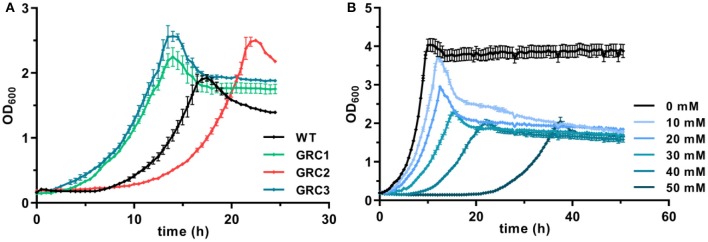
Enhanced growth of genome-reduced *Pseudomonas taiwanensis* chassis strains in the presence of *t*-cinnamate. **(A)** Growth of the *P. taiwanensis* VLB120 wildtype and the three genome-reduced chassis strains in the presence of 30 mM of *t*-cinnamate in mineral salts medium (MSM) containing 20 mM of glucose. **(B)** Growth of *P. taiwanensis* GRC3 at increasing *t*-cinnamate concentrations (0–50 mM) in MSM. Growth was monitored in the Growth Profiler®, error bars represent the standard error of the mean (*n* = 3).

These problems can be avoided by the utilization of robust, natively stress-resistant host strains such as *Pseudomonas taiwanensis* VLB120. As shown in Figure 1A, the wildtype can grow in the presence of 30 mM (4.4 g L^−1^) *t*-cinnamate in MSM with a rate of 0.24 ± 0.01 h^−1^. *P. taiwanensis* VLB120 is equipped with particular mechanisms that enable this superior tolerance toward a variety of toxic compounds. While RND-type efflux pumps mainly act on hydrophobic aromatic solvents and a number of antibiotics (Terán et al., 2003; Köhler et al., 2013; Volmer et al., 2014), an ABC transporter (Ttg2ABC) was recently identified as crucial for *p*-coumarate tolerance, the hydroxylated derivative of *t*-cinnamate (Calero et al., 2018). Furthermore, chaperone upregulation is observed as response to protein misfolding (Segura et al., 2012).

To further exploit the native potential of *P. taiwanensis* VLB120 as microbial cell factory, we recently engineered a genome-reduced variant of this strain which exhibited higher growth rates and enhanced biomass formation (Wynands et al., 2019). The benefit of genome reduction for potential aromatics production strains is underlined by the tolerance test in the presence of 30 mM *t*-cinnamate (Figure 1A). The streamlined strains GRC1 and GRC3 had a reduced lag phase in the presence of high *t*-cinnamate concentrations, while the growth rate remains similar to the wildtype. In addition, the GRC strains reached higher final OD_600_ values compared to the wildtype. This is particularly important to improve the efficiency of a microbial production process by reducing the overall process time and lowering the amount of substrate needed to generated and maintain the biomass. The only exception is strain *P. taiwanensis* GRC2, which grew slower and with a longer lag phase than the wildtype. In contrast to GRC1 and 3, GRC2 constitutively expresses *ttgGHI* genes that encode a solvent-efflux pump. Although this constitutive expression greatly increases the fitness of GRC2 in the presence of hydrophobic solvents such as toluene, it causes a fitness reduction in the absence of solvents (Wynands et al., 2019). This drawback is absent in the other two strains, where the whole *ttg* operon is absent (GRC1), or the regulatory genes *ttgVW* are included to induce the pump in response to solvents (GRC3). The similar performance of GRC1 and GRC3 in the presence of *t*-cinnamate indicates that this aromatic acid does not induce expression of the *ttgGHI*, nor does the TtgGHI pump contribute to cinnamate tolerance. Given this similar performance, and in light of the potential to use a *t*-cinnamate-producing strain as platform for a variety of further industrially relevant hydrophobic aromatics, the strain GRC3 was chosen as host to maintain the possibility of solvent efflux by TtgGHI. As shown in Figure 1B, *P. taiwanensis* GRC3 can grow in the presence of *t*-cinnamate concentrations of up to 50 mM (7.4 g L^−1^), which is 10-fold higher than MIC for *E. coli*. The native tolerance potential of *P. taiwanensis* VLB120 in combination with improved bioprocess features obtained by streamlining of this strain hence set an ideal starting point for an efficient microbial production process of *t*-cinnamate.

### Rational Engineering of L-phenylalanine Production

**Figure 2:**
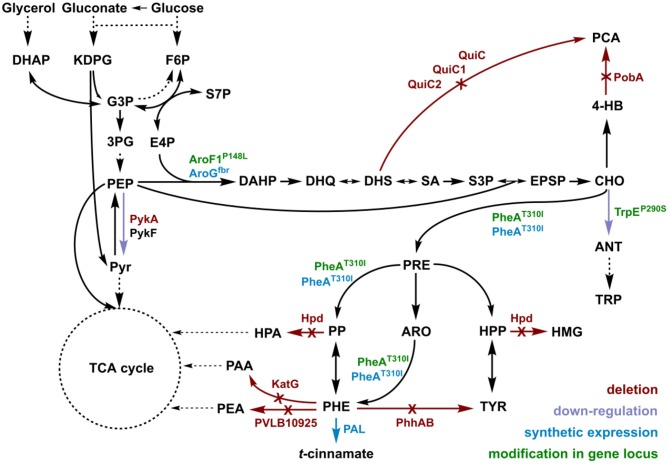
Genomic modifications introduced into strain *P. taiwanensis* GRC3 to enable accumulation of L-phenylalanine and subsequent deamination to *t*-cinnamate. Red arrows and annotations indicate gene deletions, purple arrows represent enzymatic downregulation, green annotations highlight point mutations introduced into the native gene locus, and blue tags and arrows represent the overexpression of heterologous genes. DAHP, dihydroxyacetone phosphate; KDPG, 2-keto-3-deoxy-6-phosphogluconate; F6P, fructose-6-phosphate; G3P, glyceraldehyde-3-phosphate; 3PG, 3-phosphoglycerat; S7P, seduheptulose-7-phosphate; PEP, phosphoenolpyruvate; PYR, pyruvate; E4P, erythrose-4-phosphate; DAHP, 3-deoxy-D-arabinoheptulosonate-7-phosphate; DHQ, 3-dehydroquinate; DHS, 3-dehydroshikimate; SA, shikimate; S3P, shikimate-3-phosphate; EPSP, 5-enolpyruvyl-shikimate-5-phosphate; CHO, chorismate; 4-HB, 4-hydroxybenzoate; PCA, protocatechuate; ANT, anthranilate; TRP, tryptophan; PRE, prephenate; PP, phenylpyruvate; HPP, 4-hydroxyphenylpyruvate; ARO, arogenate; TYR, tyrosine; PHE, phenylalanine; HMG, homogentisate; PAA, 2-phenylacetamide; PEA, phenylethylamine; PykA/PykF, pyruvate kinase isozymes; QuiC/QuiC1/QuiC2, 3-dehydroshikimate dehydratase isozymes; PobA, 4-hydroxybenzoate 3-monooxygenase; Hpd, 4-hydroxyphenylpyruvate dioxygenase; AroF-1^P148L^/AroG^fbr^, DAHP synthase isozymes; TrpE^P290S^, anthranilate synthase (component I); PheA^T310I^, bi-functional chorismate mutase/prephenate dehydratase; PhhAB, phenylalanine 4-monooxygenase; KatG, catalase-peroxidase; PVLB_10925, aromatic-L-amino-acid decarboxylase; PAL, phenylalanine ammonia-lyase.

**Figure 3:**
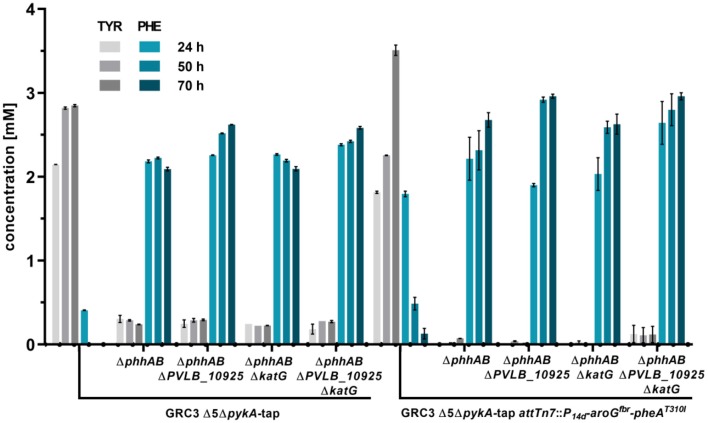
L-phenylalanine and L-tyrosine accumulation of *P. taiwanensis* GRC3 Δ5Δ*pykA*-tap and subsequent mutants with deletions to prevent L-phenylalanine degradation and additional overexpression of AroG^fbr^ and PheA^T310I^. The strains were cultivated in MSM containing 20 mM glucose in a System Duetz® shaker. Error bars represent the standard error of the mean (*n* = 3).

*t*-cinnamate is the deamination product of L-phenylalanine and the enhanced supply of this precursor in microbial chassis strains is thus crucial to allow efficient production. A variety of approaches toward L-phenylalanine overproduction were reported over the last years, delivering highly productive strains of e.g., *E. coli* (63 g L^−1^ from glucose) (Ding et al., 2016) or *Corynebacterium glutamicum* (16 g L^−1^ from glucose) (Zhang et al., 2015). Biosynthesis of L-phenylalanine and L-tyrosine both starts with the conversion of chorismate into prephenate, which then branches via phenylpyruvate to L-phenylalanine or via 4-hydroxyphenylpyruvate to L-tyrosine. Popular strategies aim to deregulate a 3-deoxy-D-arabino-heptolusonate-7-phosphate (DAHP) synthase (e.g., AroG, AroF, or AroH) or the chorismate mutase/prephenate dehydratase (PheA) activity and to increase the availability of the precursors phosphoenolpyruvate (PEP) and erythrose-4-phosphate (E4P) (Rodriguez et al., 2014; Ding et al., 2016; Huccetogullari et al., 2019). Pseudomonads have the catabolic potential to degrade a high number of aromatic compounds. This ability is a key feature applied for many biotechnological aspects such as the metabolism of complex substrates (Xu et al., 2018), but it also increases the difficulty of manufacturing aromatic amino acid accumulation in this species. We recently reported a study describing the rational engineering of *P. taiwanensis* VLB120 to produce phenol via the aromatic amino acid L-tyrosine (Wynands et al., 2018). The deletion of five genes (*pobA, hpd, quiC, quiC1, quiC2*) associated to the degradation of shikimate pathway-derived compounds resulted in strains unable to catabolize L-tyrosine, L-phenylalanine, *p*-hydroxybenzoate, and 3-dehydroshikimate to ensure aromatics accumulation. The flux toward L-tyrosine was subsequently enhanced by the insertion of point mutations into the native locus of genes in the shikimate pathway, resulting in enzymes with amino acid substitutions (TrpE^P290S^, AroF-1^P148L^, PheA^T310I^). The deletion of *pykA* (encoding for a pyruvate kinase) additionally reduced the flux of PEP toward pyruvate, thereby increasing the precursor pool for the shikimate pathway. An introduction of these mutations in the genome-reduced strains of *P. taiwanensis* VLB120 led to the efficient production of phenol from L-tyrosine (Wynands et al., 2019). As shown in Figure 3, the strain *P. taiwanensis* GRC3 Δ5Δ*pykA*-tap accumulates 2.85 ± 0.02 mM of tyrosine from 20 mM of glucose, which is comparable to the non-genome-reduced strain. Three genes involved in phenylalanine catabolism were subsequently deleted from the chromosome of *P. taiwanensis* GRC3 Δ5Δ*pykA*-tap: (i) *phhAB* encoding for phenylalanine-4-monooxygenase involved in the conversion of phenylalanine to tyrosine (Herrera et al., 2010), (ii) PVLB_10925, putatively encoding an aromatic-L-amino-acid decarboxylase responsible for the decarboxylation into phenylethylamine, and (iii) *katG*, putatively coding for a catalase-peroxidase which converts phenylalanine into phenyl acetamide (Figure 2).

Pseudomonads possess a variety of transporters that allow export and import of aromatic amino acids. Transcriptome analysis of a *Pseudomonas putida* strain with increased flux toward tyrosine revealed that upon increased intracellular aromatic amino acid levels, amino acid exporters are upregulated, while uptake systems are downregulated (Wierckx et al., 2008). Aromatics accumulation shown in Figure 3 displays extracellular concentrations, indicating enhanced efflux of both tyrosine and phenylalanine.

The deletion of *phhAB* led to the accumulation of 2.22 ± 0.02 mM of phenylalanine and 0.29 ± 0.01 mM tyrosine after 50 h (Figure 3). This indicates that about 88% of L-tyrosine accumulating due to the enhanced flux toward prephenate in strain *P. taiwanensis* GRC3 Δ5Δ*pykA*-tap is stemming from L-phenylalanine. In most microorganisms, L-phenylalanine cannot be converted into L-tyrosine in which case it is solely synthesized from prephenate (Guroff and Ito, 1965). In *Pseudomonas* however, the pathway via L-phenylalanine seems to be a major route for L-tyrosine formation under the given conditions (Wierckx et al., 2009). As described for other *Pseudomonas* species, Xanthomonads and *Alcalignes* (Ahmad et al., 1990), this diversity of aromatic amino acid metabolism account for the flexibility of the organism to cope with a variety of end product analogs (Fiske et al., 1983). The concentration of L-phenylalanine in the culture of *P. taiwanensis* GRC3 Δ5Δ*pykA*-tapΔ*phhAB* was slightly reduced after 70 h, indicating that other pathways involved in L-phenylalanine degradation were still active in this strain. The subsequent deletion of gene PVLB_10925 prevented this degradation, resulting instead in a further increase of the final titer to 2.62 ± 0.00 mM L-phenylalanine. The deletion of *katG* had no influence on phenylalanine accumulation and the strain shows a similar production pattern as the strain without a deletion. Indeed, as the progenitor strain *P. taiwanensis* GRC3 Δ5Δ*pykA*-tap was already not able to grow on L-phenylalanine and L-tyrosine (Wynands et al., 2018), this confirms that catabolic pathways are either not active (*katG*) or not connected to the central carbon metabolism (PVLB_10925) under the applied conditions, even though this strain contains the genetic inventory for the full degradation of the resulting 2-phenylacetamide and phenylethylamine. This is underlined by similar observations in an L-phenylalanine overproducing strain of *P. putida* DOT-T1E (Molina-Santiago et al., 2016).

L-Tyrosine and L-phenylalanine production could be increased further by the additional overexpression of AroG^fbr^, a feedback inhibition resistant versions of the DAHP synthase from *E. coli* K12 W3110 (Kikuchi et al., 1997) and PheA^T310I^ from *P. putida* S12palM12 (Nijkamp et al., 2005). The coding genes were integrated chromosomally at the *attTn7*-site and expressed under the control of the constitutive promoter P_14g_ (Zobel et al., 2015). Overexpression in the tyrosine-accumulating strain *P. taiwanensis* GRC3 Δ5Δ*pykA*-tap led to tyrosine titers of about 3.5 ± 0.11 mM after 70 h. Transient L-phenylalanine accumulation was observed with this strain, further underlining the high flux through PhhAB. Upon overexpression of *aroG*^fbr^ and *pheA*^T310I^ in strains with deletions of *phhAB, katG* and PVLB_10925, the resulting strain GRC3 Δ8Δ*pykA*-tap *attTn7*::*P*_14*d*_-*aroG*^fbr^-*pheA*^AT31I^ (Δ8 = eight deletions of pathways involved in aromatics degradation) accumulated 3.0 ± 0.07 mM of L-phenylalanine after 70 h. Also, L-tyrosine production was reduced, likely due to PheA^T310I^ increasing the flux of prephenate toward L-phenylalanine.

### Production of *t*-cinnamate From Glucose and Glycerol in Shake Flasks

**Figure 4:**
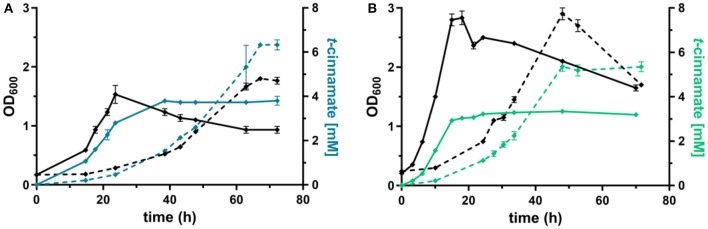
Shake flask cultivations of *P. taiwanensis* GRC3 Δ8Δ*pykA*-tap with varying heterologous expression modules. **(A)** Growth (black lines) and t-cinnamate production (blue lines) by *P. taiwanensis* GRC3 Δ8Δ*pykA*-tap *attTn7*::*P*_14*g*_*AtPAL- aroG*^*fbr*^*-pheA*^*T*310^ in MSM containing 20 mM glucose (solid line) or 40 mM of glycerol (dotted line). **(B)** Growth (black lines) and *t*-cinnamate production (green lines) by *P. taiwanensis* GRC3 Δ8Δ*pykA*-tap *attTn7*::*P*_14*f*_*AtPAL* in MSM containing 20 mM glucose (solid line) or 40 mM of glycerol (dotted line). Error bars represent the standard error of the mean (*n* = 3).

The L-phenylalanine-accumulating strain *P. taiwanensis* GRC3 Δ8Δ*pykA*-tap was subsequently used as host for the production of *t*-cinnamate. The deamination of L-phenylalanine into *t*-cinnamate was achieved using the PAL2 enzyme from *A. thaliana*. While four genes encoding PAL have been identified in *A. thaliana* (Cochrane et al., 2004), PAL2 displayed the highest specific activity when expressed in *E. coli* (McKenna and Nielsen, 2011). In addition, it has no activity on L-tyrosine as a substrate, in contrast to a variety of other yeast PALs (Cui et al., 2008). As overexpression of *aroG*^fbr^ and *pheA*^T310I^ improved L-phenylalanine yields by 15% (Figure 3), combinatorial expression with PAL on the *Tn7* transposon under the control of P_14g_ was applied for *t*-cinnamate production in the strain *P. taiwanensis* GRC3 Δ8Δ*pykA*-tap *attTn7*::*P*_14*g*_*AtPAL*- *aroG*^fbr^-*pheA*^T310^. The strain was cultivated in shake flasks in MSM containing either 20 mM of glucose or 40 mM of glycerol as sole carbon source. Glycerol is a promising alternative as feedstock for microbial production processes as abundant by-product of the bio-diesel production (Zambanini et al., 2016). When cultivated on glucose, the strain accumulated 3.80 ± 0.20 mM *t*-cinnamate, corresponding to a yield of 25.9 ± 0.1% Cmol Cmol^−1^, with a volumetric productivity of 0.10 ± 0.00 mM h^−1^ (Figure 4). From glycerol, the *t*-cinnamate titer was further increased to 6.33 ± 0.12 mM, corresponding to a yield of 47.5 ± 0.9% Cmol Cmol^−1^. As observed for the production of phenol and *p*-hydroxybenzoate (Wynands et al., 2018; Lenzen et al., 2019), metabolism of glycerol is highly beneficial for enhanced titers and yields of compounds derived from aromatic amino acids, likely due to metabolic rearrangements favoring the supply of PEP and E4P (Nikel et al., 2014; Poblete-Castro et al., 2019). The volumetric productivity on glycerol is slightly lower compared to glucose (0.09 ± 0.01 mM h^−1^), as a result of the reduced growth rate. While this strain has a very high yield, its reliable application as a production host was problematic. When the experiment described above was repeated, the cultures displayed varying growth behavior in terms of lag-phase, growth rates, and productivity (Figure S1), even though the cultivation procedure remained identical. Re-transformation of the Tn7-transposon bearing the overexpression constructs and culturing of single colonies yielded the same unstable phenotype. Sequencing of the *attTn7*-regions of theses strains revealed no mutations in this sequence and verified correct integration. Likely, a combination of the extreme drain on carbon posed by the high *t*-cinnamate yield, the burden of heterologous overexpression of genes, and the production of a mildly toxic compound provide a strong selection for suppressor mutations. This impairs the reproducibility and renders the strain *P. taiwanensis* GRC3 Δ8Δ*pykA*-tap *attTn7*::*P*_14*g*_*AtPAL*- *aroG*^fbr^-*pheA*^T310^ unsuitable as reliable production host in its current form.

In contrast, *P. taiwanensis* GRC3 Δ8Δ*pykA*-tap *attTn7*::*P*_14*f*_*AtPAL*, harboring only the PAL for L-phenylalanine deamination under the control of the weaker P_14f_ promoter, showed reliable growth and production patterns. This strain still carries feedback-resistant *aroF-1*^*P*148*L*^*, pheA*^*T*310*I*^, but only in their native context. The lack of *in trans* overexpression of *aroG*^fbr^ and *pheA*^T310I^ reduced *t*-cinnamate titers and yields slightly, with 3.34 ± 0.07 mM produced from glucose and 5.25 ± 0.22 mM from glycerol, which corresponds to yields of 22.8 ± 0.5 and 38.9 ± 1.6% Cmol Cmol^−1^, respectively. To the best of our knowledge, these are still the highest yields of *t*-cinnamate produced in a microbial process using a mineral medium without the addition of complex supplements. This strain furthermore reached higher final biomass and exhibited improved volumetric productivity on glucose (0.13 ± 0.00 mM h^−1^) and glycerol (0.11 ± 0.00 mM h^−1^) compared to the strain additionally expressing *aroG*^fbr^ and *pheA*^T310I^. In the course of the cultivations, no accumulation of L-phenylalanine was observed. This indicates that PAL activity is not inhibited by the concentrations of *t*-cinnamate reached in these cultures, in contrast to observations in other hosts and with PALs from different organisms (Nijkamp et al., 2005; McKenna and Nielsen, 2011; Molina-Santiago et al., 2016). Furthermore, no L-tyrosine accumulation was observed, in contrast to the phenylalanine-producing equivalent that does not express PAL (Figure 3), likely due to a reduction of product inhibition of the upstream metabolic enzymes as a result of efficient conversion to *t*-cinnamate.

### Production in Controlled Bioreactors

In order to assess higher-level *t*-cinnamate production under more industrially relevant conditions, the strains were cultivated in fed-batch fermentations in controlled bioreactors. The fermenters were operated in a dO_2_-stat fed-batch mode where carbon depletion and the resulting increase of the dO_2_ due to metabolic arrest triggered the initiation of a pulse feed of either 5 mM glucose or 10 mM glycerol (Johnson et al., 2016, 2019). This feeding protocol allows controlled addition of carbon source throughout the whole fermentation process, independent from time-varying carbon demands of the cell, thereby preventing excess carbon surplus (Johnson et al., 2016). The resulting feeding trends of single reactors are exemplarily shown in Figure S3. Cultivation took place at pH 7 in a mineral medium without the addition of complex supplements.

**Figure 5:**
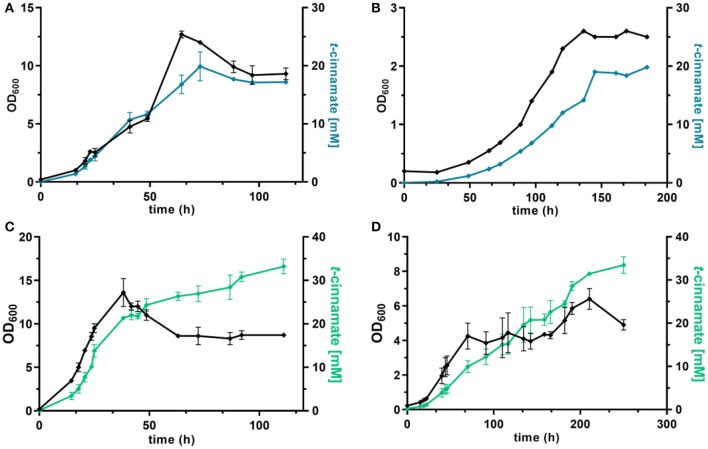
dO_2_-stat fed-batch fermentations of *t*-cinnamate producing strains of *P. taiwanensis*. Cultivations were performed in MSM, where the initial batch medium contained either glucose or glycerol as sole carbon source and the subsequent feeding solution contained solely the respective carbon source. The top graphs show growth (black lines) and *t*-cinnamate accumulation (blue lines) during fermentation of *P. taiwanensis* GRC3 Δ8Δ*pykA*-tap *attTn7::P*_14*g*_*AtPAL- aroG*^*fbr*^*-pheA*^*T*310^ on glucose **(A)** and glycerol **(B)**. The glycerol figure **(B)** displays data of a single reactor. The bottom graphs represent growth (black lines) and *t*-cinnamate accumulation (green lines) during fermentation of *P. taiwanensis* GRC3 Δ8Δ*pykA*-tap *attTn7::P*_14*f*_*AtPAL* on glucose **(C)** and glycerol **(D)**. The error bars represent the standard error (*n* = 2).

Under these conditions, the strain *P. taiwanensis* GRC3 Δ8Δ*pykA*-tap *attTn7*::*P*_14*g*_*AtPAL*-*aroG*^fbr^-*pheA*^T310^ accumulated 17.2 ± 0.28 mM of *t*-cinnamate from glucose, corresponding to a yield of 11.2 ± 0.6% (Cmol Cmol^−1^) and a volumetric productivity of 0.19 ± 0.20 mM h^−1^. As shown in Figure 5A, the culture reaches a final OD_600_ of 12.7 after around 65 h. At this point, a decrease in biomass concentration was observed. Furthermore, there was no significant increase in *t*-cinnamate titers from this point on, in spite of the fact that the base strain GRC3 tolerated higher concentrations of *t*-cinnamate up to 50 mM (Figure 1). It is possible that several factors impact the lowered growth performance of the production strains in the presence of *t*-cinnamate. One factor is the overexpression of heterologous genes which leads to lowered growth rates and impaired fitness as observed in shake flask experiments (Figure 4). Especially the overexpression of AroG and PheA appears to lead to increased cellular stress, which in turn also lowers the growth performance in the presence of toxic compounds. Furthermore, experiments regarding styrene toxicity have demonstrated a crucial difference in tolerance depending on whether a compound is added exogenously to the medium or produced intracellularly (Lian et al., 2016).

Fed-batch fermentations using glycerol as carbon source further underline complications using the phenotypically unstable strain *P. taiwanensis* GRC3 Δ8Δ*pykA*-tap *attTn7*::*P*_14*g*_*AtPAL*-*aroG*^fbr^-*pheA*^T310^ (Figure 5B; Figure S2). During fermentations, cultures in duplicate reactors showed different growth behavior while stemming from the same pre-culture. The cultures showed differences in lag-phase, growth rate and productivity. The final titers remained comparable, likely as a result of product inhibition as described before. Figure 5B shows a single fermentation on glycerol as an example. Here, the strain produced up to 19.8 mM *t*-cinnamate, with a yield of 47.8% Cmol and a volumetric productivity of 0.13 mM h^−1^. The extended lag phase observed during glycerol assimilation (Poblete-Castro et al., 2019) might even increase the occurrence of suppressor mutations by induction of the SOS response.

As observed for shake flask cultivations, reproducibility and stable production was restored in a strain overexpressing solely PAL for L-phenylalanine conversion. The strain *P. taiwanensis* GRC3 Δ8Δ*pykA*-tap *attTn7*::*P*_14*f*_*AtPAL* produced up to 33.2 ± 2.4 mM *t*-cinnamate from glucose (Figure 5C) and 33.5 ± 2.7 mM from glycerol (Figure 5D) in a fed-batch fermentation. While the productivity throughout the whole cultivation of *P. taiwanensis* GRC3 Δ8Δ*pykA*-tap *attTn7*::*P*_14*f*_*AtPAL* on glucose was 0.30 ± 0.02 mM h^−1^, increasing concentrations of *t*-cinnamate impaired the strain's fitness and productivity. The volumetric productivity within the first 25 h is 0.55 ± 0.08 mM h^−1^, which is reduced after 38 h to 0.16 ± 0.04 mM h^−1^. At the same time, a drop in OD_600_ was observed at concentrations above 20 mM *t*-cinnamate. As production continued and cells were still viable at this point as indicated by the ongoing substrate consumption, this drop in OD_600_ values hints toward an increased cumulative burden on the cell. The drop in OD_600_ was not caused by biofilm formation, likely due to the deletion of the biofilm-associated *lap* genes in the GRC3 strain. Membrane adaptation is a key mechanisms of *Pseudomonas* to cope with environmental stress. Membrane active substances and certain environmental conditions cause adaptations such as *cis*- to *trans*-isomerization of fatty acids to increase membrane rigidity and lead to the formation of outer membrane vesicles to facilitate biofilm formation (Eberlein et al., 2018). In addition, many porins as well as import and export pumps are differentially expressed under stress, altering membrane permeability (Volkers et al., 2006; Ramos et al., 2015). The importance of membrane structure on similar compounds such as *p*-coumarate has recently been demonstrated (Calero et al., 2018) and can likely be partially be transferred to *t*-cinnamate tolerance.

As enzyme inhibition of different PALs by *t*-cinnamate has been observed in various studies (Nijkamp et al., 2007; McKenna and Nielsen, 2011), the decrease in productivity could also be linked to this effect. However, no L-phenylalanine accumulation was observed over the course of the fermentations, indicating that the precursor was completely converted by the PAL. Furthermore, no L-tyrosine accumulation was observed during fermentations.

In contrast to the strain overexpressing AroG^fbr^ and PheA^T310I^, experiments with strain *P. taiwanensis* GCR3 Δ8Δ*pykA*-tap *attTn7*::*P*_14*f*_*AtPAL* are highly reproducible. While an additional expression of AroG^fbr^ and PheA^T310I^ led to higher yields in shake flasks, solely PAL expression in the L-phenylalanine-overproducing chassis strains enables both higher titers and volumetric productivities in fed-batch fermentations (Table 3). To the best of our knowledge, these are the highest *t*-cinnamate yields reported for a microbial production process. While titers of 6.5 g L^−1^ (43.9 mM) have been reported in fed-batch fermentations of *t*-cinnamate-producing strains of *E. coli* (Bang et al., 2018), these processes required the addition of yeast extract in the initial batch medium and casamino acid in the feeding solution. The utilization of these engineered *P. taiwanensis* GRC strains enables growth without the addition of complex supplements. However, tolerance mechanisms for *t*-cinnamate remain to be further investigated and exploited to enhance titers and thus the feasibility of microbial *t*-cinnamate synthesis.

**Table 3:** Comparison of *t*-cinnamate titer, yield, and volumetric productivity of strains *P. taiwanensis* GRC3 Δ8Δ*pykA*-tap *attTn7*::P_14g_AtPAL- aroG^fbr^-pheA^T310^ and GRC3 Δ8Δ*pykA*-tap *attTn7*::*P*_14*f*_*AtPAL* in shake flask cultivations and fed-batch fermentations.

		**GCR3** **Δ8Δ*****pykA trpE***^****P290S****^ ***aroF-1***^****P148L****^ ***pheA***^****T310I****^ **(tap)**	
		***attTn7*::*P_**14*g***_AtPAL*-*aroG*^**fbr**^-*pheA*^**T310**^**	***attTn7*::*P_**14*f***_AtPAL***
Shake flask glucose	Final titer [mM]	3.8 ± 0.20	3.3 ± 0.07
	Yield (% Cmol Cmol^−1^)	25.9 ± 0.1	22.8 ± 0.5
	Productivity (mM h^−1^)	0.10 ± 0.00	0.13 ± 0.00
Shake flask glycerol	Final titer [mM]	6.3 ± 0.12	5.4 ± 0.22
	Yield (% Cmol Cmol^−1^)	47.5 ± 0.9	38.9 ± 1.6
	Productivity (mM h^−1^)	0.09 ± 0.01	0.11 ± 0.00
Fed-batch glucose	Final titer [mM]	17.2 ± 0.3	33.2 ± 2.4
	Yield (% Cmol Cmol^−1^)	11.2 ± 0.6	21.4 ± 1.1
	Productivity (mM h^−1^)	0.19 ± 0.20	0.30 ± 0.02
Fed-batch glycerol	Final titer [mM]	19.8 (*n* = 1)	33.5 ± 2.7
	Yield (% Cmol Cmol^−1^)	47.8 (*n* = 1)	36.1 ± 0.08
	Productivity (mM h^−1^)	0.13 (*n* = 1)	0.15 ± 0.00

## Conclusion

In this study, we describe the rational engineering of *P. taiwanensis* VLB120 towards efficient *t*-cinnamate production. The plasmid-free strain bearing no auxotrophies synthesized *t*-cinnamate from glucose or glycerol, with yields of up to 48% Cmol Cmol^−1^ in shake flask cultivations. Titers were increased up to 33.35 mM in fed-batch fermentations using glycerol as sole carbon source. As product titers achieved in this study impair fitness and productivity of the chassis strains, the native tolerance features of *Pseudomonas* allowing enhanced *t*-cinnamate tolerance require further investigation and enhancement to increase process efficiency. One promising target is the ABC transporter Ttg2ABC, an extrusion pump involved in *p*-coumarate tolerance (Calero et al., 2018). An overexpression of this pump might deliver enhanced tolerance toward *t*-cinnamate, its regulation however remains to be investigated. Fine-tuning of additional *aroG* and *pheA* overexpression, e.g., by using weaker or inducible promoters, could furthermore avoid the observed growth defects while still maintaining the high yields achieved with this setup. Overall, the results underline the high potential of *Pseudomonas* species to produce chemical building blocks using aromatic amino acids as precursors. The establishment of efficient microbial production of the model compound *t*-cinnamate will in future serve as foundation to expand the product collection of this versatile species, ranging from bulk chemicals such as styrene (Lee et al., 2019) to specialty compounds such as stilbenes (van Summeren-Wesenhagen and Marienhagen, 2015).

## Data Availability Statement

All datasets generated for this study are included in the article/Supplementary Material.

## Author Contributions

NW conceived and supervised the study with the help of LB. MO and MF performed the experiments with support of BW and CL. BW provided the GRC strains with deletions for L-tyrosine accumulation and deletion vectors. MO wrote the manuscript with the help of BW and NW. All authors read and approved the manuscript.

### Conflict of Interest

The authors declare that the research was conducted in the absence of any commercial or financial relationships that could be construed as a potential conflict of interest.
