# Supplementary material for: Rational Engineering of Phenylalanine Accumulation in Pseudomonas taiwanensis to Enable High-Yield Production of Trans-Cinnamate
Source: Front Bioeng Biotechnol. 2019 Nov 20;7:312. doi: 10.3389/fbioe.2019.00312 (PMC6882275; doi:10.3389/fbioe.2019.00312)
Supplement: Supplementary file 1 [file Data_Sheet_1.docx]

**Supplementary Material**


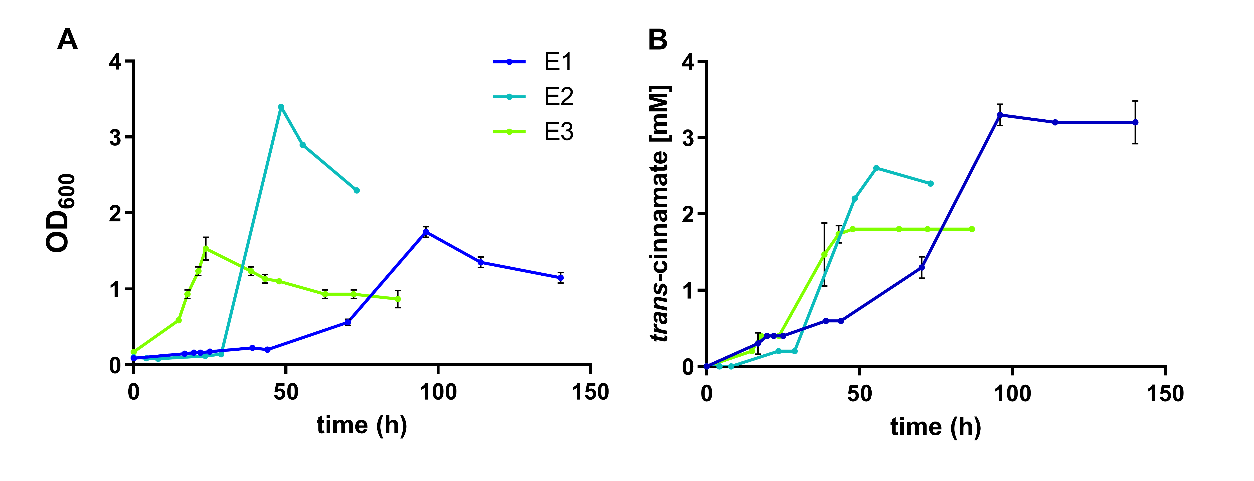


Figure S1. Growth (A) and *t*-cinnamate accumulation (B) during shake flask cultivations of strain *P. taiwanensis* GRC3 ∆8∆*pykA*-tap *attTn7*::*P_14g_AtPAL- aroG^fbr^-pheA^T310^* in MSM containing 20 mM of glucose. The graphs represent the data of three individual experiments (E1, E2, E3) performed on different days after an identical pre-culturing procedure. Error bars represent the standard error of the mean (n=3 for E1; n=2 for E2 and E3).


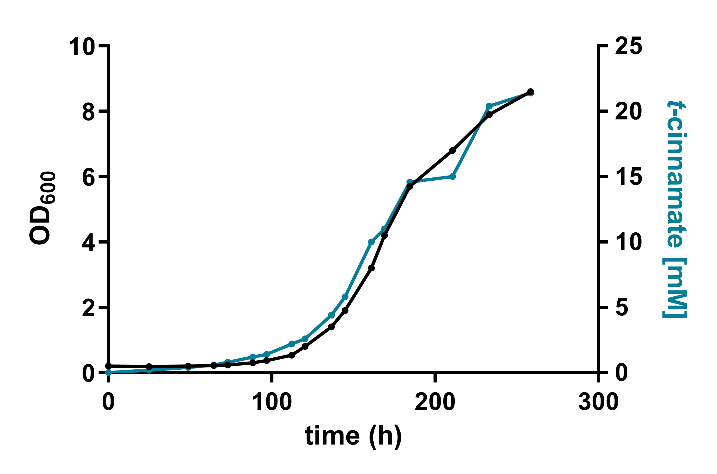


Figure S2. dO_2_-stat fed-batch fermentation of *P. taiwanensis* GRC3 ∆8∆pykA-tap *attTn7*::P*_14g_AtPAL- aroG^fbr^-pheA^T310^* using glycerol as sole carbon source. Cultivation was performed in MSM, where the initial batch medium and the subsequent feeding solution contained solely glycerol as carbon source. Growth (black lines) and *t*-cinnamate accumulation (blue lines) of a single reactor is shown, which was performed simultaneously with the single reactor shown in Figure 5B.


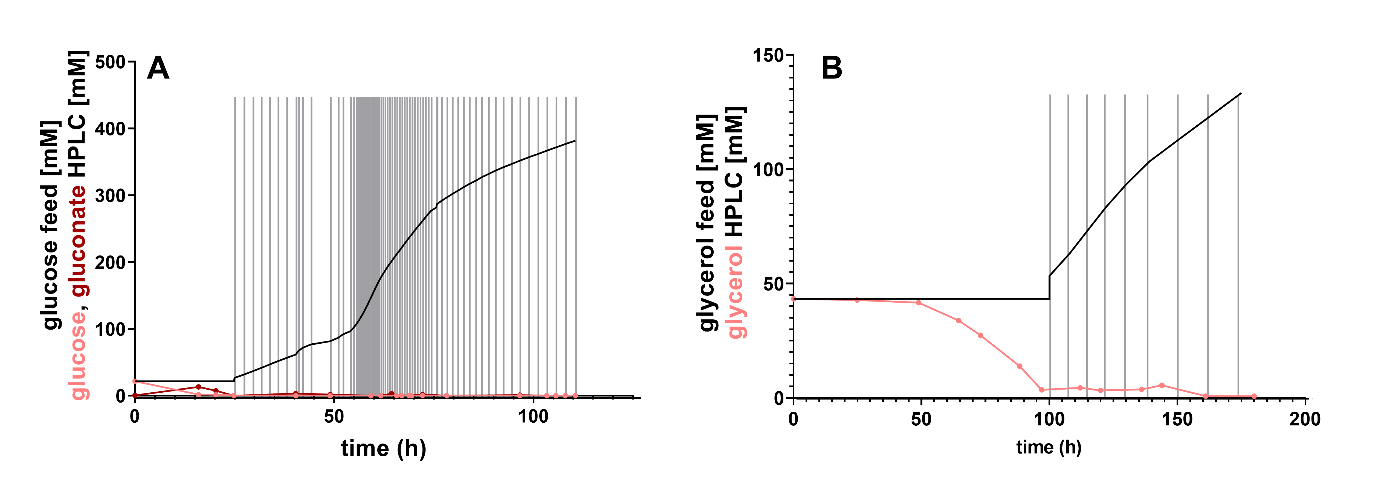


Figure S3. Examples of single dO_2_-stat fed-batch fermentations of *P. taiwanensis* GRC3 ∆8∆*pykA*-tap *attTn7*::*P_14g_AtPAL- aroG^fbr^-pheA^T310^* using either glucose (A) or glycerol (B) as sole carbon source. The gray bars represent one feed pulse triggered by rising dO_2_-signals as a result of carbon depletion. The black lines indicate the cumulative amount of carbon source fed, red/pink symbols represent HPLC measurements.
